# Differences between panoramic and Cone Beam-CT in the surgical evaluation of lower third molars

**DOI:** 10.4317/jced.53234

**Published:** 2017-02-01

**Authors:** Ruggero Rodriguez y Baena, Riccardo Beltrami, Angelo Tagliabo, Silvana Rizzo, Saturnino-Marco Lupi

**Affiliations:** 1 Prof, MD. University of Pavia, Department of Clinical Surgical, Diagnostic and Pediatric Sciences, P.le Golgi 2, 27100, Pavia, Italy; 2 PhD, DDS. University of Pavia, Department of Clinical Surgical, Diagnostic and Pediatric Sciences, P.le Golgi 2, 27100, Pavia, Italy; 3 MD. University of Pavia, Department of Clinical Surgical, Diagnostic and Pediatric Sciences, P.le Golgi 2, 27100, Pavia, Italy

## Abstract

**Background:**

The aim of this study was to evaluate the ability to identify the contiguity between the root of the mandibular third molar and the mandibular canal (MC) in panoramic radiographs compared with Cone Beam-CT.

**Material and Methods:**

Panoramic radiographs of 326 third molars and CBCT radiographs of 86 cases indicated for surgery and considered at risk were evaluated. The following signs were assessed in panoramic radiographs as risk factors: radiolucent band, loss of MC border, change in MC direction, MC narrowing, root narrowing, root deviation, bifid apex, superimposition, and contact between the root third molar and the MC.

**Results:**

Radiographic signs associated with absence of MC cortical bone are: radiolucent band, loss of MC border, change in MC direction, and superimposition. The number of risk factors was significantly increased with an increasing depth of inclusion. CBCT revealed a significant association between the absence of MC cortical bone and a lingual or interradicular position of the MC.

**Conclusions:**

In cases in which panoramic radiographs do not exclude contiguity between the MC and tooth, careful assessment the signs and risks on CBCT radiographs is indicated for proper identification of the relationships between anatomic structures.

** Key words:**Panoramic radiography, Cone-Beam computed tomography, third molar, mandibular nerve.

## Introduction

Extraction of the third molars is the most commonly performed oral surgery (1) and surgical extraction of the mandibular third molars is the leading cause of injury to the inferior alveolar nerve (IAN), (2,3) which can be temporary or permanent (4). The neurological injury is often associated with malpractice litigation and request for compensation (5). Almost 80% of oral and maxillofacial surgeons in California know of patients with permanent injury to the IAN following the removal of a third molar (6); in Finland, in 1987-1993, 2% of dentists and 26% of oral surgeons faced claims for injuries caused by the extraction of third molars (3). Based on the literature, the incidence of injury to the IAN varies from 0.4% to 22% (4,5,7).

The risk of injury is increased when the root of the third molar is in close proximity to the mandibular canal (MC) (7,8), and therefore proper presurgical planning is required to reduce the risk of injury to the IAN (9,10). Third molars are difficult to evaluate, however, and panoramic radiography is routinely used (9-11). Some previous studies have identified panoramic signs that indicate a close anatomic correlation between the root of the third molar and the MC, which is thought to increase the risk of neurological injury (9,12).

Panoramic radiography is limited because it provides a two-dimensional (2D) image of three-dimensional (3D) structures, with distortion of the linear measures and a loss of definition due to the superimposition of underlying structures (13). Thus, the borders of the MC cannot be clearly distinguished in every panoramic exam (14). As a result, assessment of panoramic radiographs by different observers may lead to different results (12). In cases with signs of contiguity between the MC and the root of the third molar, further investigation with a 3D X-ray may be appropriate (12).

CBCT radiography is indicated in these cases due to its diagnostic capacity and reduced radiation dose administered to the patient, corresponding to 3% to 20% of the dose of a traditional CT, and comparable to the dose of 2D X-ray (10).

The aim of the present study was to evaluate the relationship between the root of mandibular third molars and the MC in patients who underwent examination for third molar tooth extraction. The panoramic signs of the relationship between the third molars and MC were evaluated and correlated with those of CBCT to assess their adequacy for identifying a true absence of MC cortical bone.

## Material and Methods

All X-rays of patients visiting the Dental Service of the Department of Clinical Surgical, Pediatric Diagnostics at the University of Pavia between September 2013 and May 2015 for extraction of a mandibular third molar were evaluated. All patients underwent clinical examination and panoramic radiography. In cases with clinical indications for extraction and radiographic signs of contiguity between the roots of the third molar and MC according to Koong and colleagues (11), the patients underwent CBCT.

Digital panoramic radiographs were obtained with a Soredex CRANEX™ D device (Soredex, Helsinki, Finland), set at 73 kV, 10 mA, 6.17 s, using a photostimulable phosphor plate. The mandibular CBCT scan was acquired using a Soredex SCANORA™ 3D device (Soredex, Helsinki, Finland; Receptor type: CMOs flat panel 124 mm x 124 mm; fixed anode tube; focal spot 0.5 mm IEC 60336; 85 kV; 4.0-12.5 mA; voxel sizes 0.25mm; scan time 13 s).

The CBCT and panoramic images were analyzed in random order by two independent and blinded observers on a 27” iMac® (Apple, Cupertino, CA, USA) monitor in a darkened room. In case of disagreement between the two observers, a consensus was reached by discussion.

CBCT radiographs were shown to the observers 1 month after the panoramic X-Rays in a different and random order. Each panoramic image or series of CBCT images was identified by a blinded code that permitted the association between the CBCT and panoramic images of the same patient after assessment of the signs, before the statistical analysis. Data were collected using a spreadsheet format on Excel® (Microsoft ® Excel ® 2011, Version 14.1.0). Digital panoramic images were evaluated by dedicated software (Digora ™ 2.6, Soredex, Helsinki, Finland).

In panoramic radiographs, the following signs were evaluated as dichotomous according to the literature (11): radiolucent band, loss of MC border, change in MC direction, MC narrowing, root narrowing, root deviation, bifid apex, superimposition between the third molar root and the MC, and contact between the third molar root and the MC.

Moreover, the position and the degree of inclusion of the third molar were evaluated in panoramic radiographs using the classification of Pell & Gregory (15), in which classes I, II and III are associated respectively to a progressive reduction of the space between the distal surface of the second molar and the anterior border of the ramus of the mandible, and classes A, B, and C respectively indicate a greater depth of inclusion than the occlusal plane.

Contiguity in the coronal, sagittal, and transverse planes was evaluated in CBCT images using SimPlant Pro 16.0 (Materialise Dental, Leuven, Belgium). In addition, the panoramic plane was rebuilt and orthogonal images to that plane were evaluated. In sections where these planes appeared minor, the distances were measured.

In CBCT, the following parameters were evaluated: the position of the MC with respect to the third molar classified as lingual, buccal, interradicular, or inferior (12); the presence/absence of cortical bone between the root of the third molar and MC (dichotomous variable); and the minimum distance between the MC and the root of the third molar (continuous variable). The distance is reported as a whole number in millimeters. Therefore, in cases in which the MC cortical bone was present and the distance reported was 0 mm, the actual distance was <0.5 mm.

-Statistical analysis

Descriptive statistics of patient variables, tooth location, radiological parameters of the relationship of the MC and third molars, position of third molars in CBCT, and distance to the mandibular nerve were calculated.

The frequencies of the nine radiological parameters were calculated in the panoramic and CBCT exams and a new variable was created to register all the parameters for each tooth.

Pell & Gregory classes were merged to increase to the number of observations in each class and strengthen the statistical analyses, as in previous research (16). The clinical concept was to merge the classes in which the third molar was rated at the same occlusal level, thus obtaining three classes: tooth at the occlusal third, tooth at the intermediate third, and tooth at the apical third.

Fisher’s exact test was used to assess the relation between the three classes and the nine parameters. The chi-square test was used to assess the relation between the nine Pell & Gregory classes and the prescription for CBCT to investigate potential correlations. Fisher’s exact test was used to assess whether the nine parameters in the panoramic radiographs were related to an interruption in the MC cortical bone in CBCT. The correlation between the position of the canal and tooth with the presence/absence of an interruption of the MC cortical bone was investigated. Statistical analysis was performed using StataCorp. 2011 (Stata Statistical Software: Release 12. College Station, TX: StataCorp LP).

## Results

The study comprised 326 mandibular third molars (167 left, 159 right) of 257 patients (132 females, 125 males). According to Pell & Gregory classification the molars included in class A1 are 116 (35.58%), 50 (15.34%) in A2, 5 (1.53%) in A3, 21 (6.44%) in B1, 47 (14.42%) in B2, 22 (6.75%) in B3, 13 (3.99%) in C1, 22 (6.75%) in C2, 25 (7.67%) in C3. Five of 326 records were not categorized because of unclear radiographs. The frequencies of radiological parameters in the panoramic exam and CBCT were calculated and are reported in  Table 1.

**Table 1:**
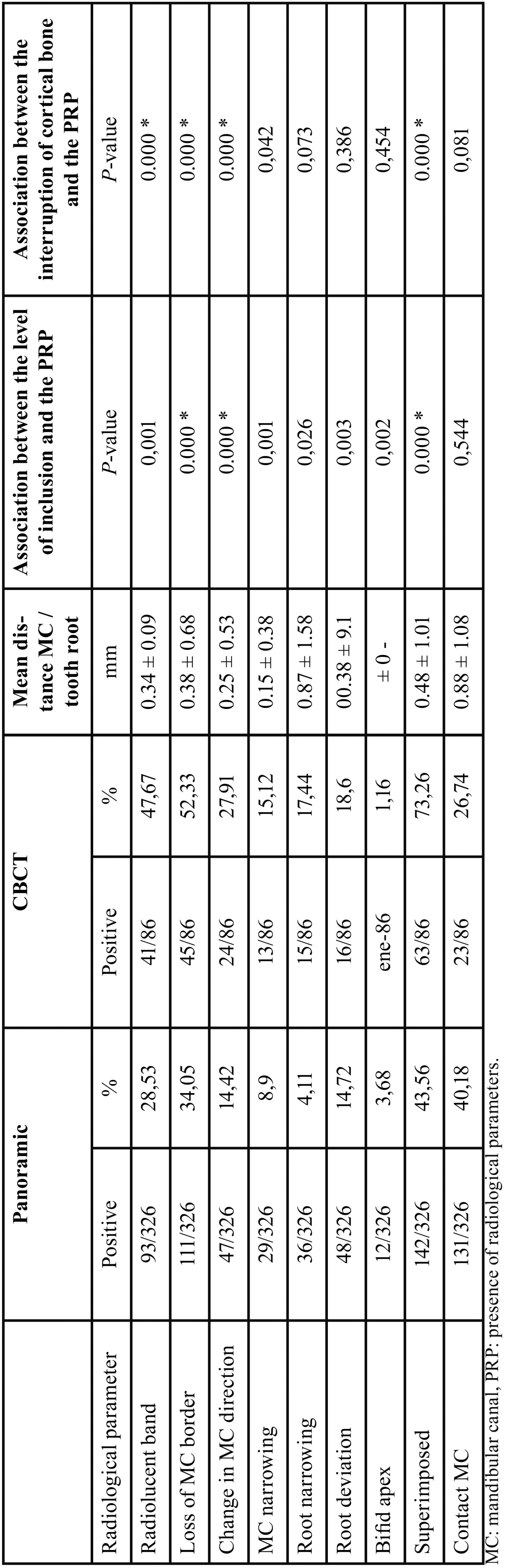
Radiological parameters for panoramic radiograph and CBCT and their association to level of inclusion and interruption of cortical bone.

All patients underwent digital panoramic radiographs, and CBCT was indicated for 86 cases.

In the panoramic radiographs, 71 teeth (21.78%) presented with none of the 9 parameters and 1 tooth (0.31%) with 7. Of the 255 teeth positive for at least one panoramic radiological parameter, only 86 third molars were indicated for surgery, thus requiring CBCT. Radiological parameters were variably distributed between the levels of inclusion of the third molars; in particular, a high significant association was recorded for loss of the MC border, change in MC direction, MC narrowing, and superimposition of the nerve and tooth (*P* < 0.0001). The frequencies of positive radiological parameters were lower for teeth with a lower level position. The chi-square test confirmed a relation between the prescription for the CBCT and the Pell & Gregory classification (*P* <0.001). CBCT was required mainly when there was MC narrowing, root deviation, bifid apex, and the tooth and nerve were superimposed.

Interruption of the MC cortical bone was detected in 44 cases of 86 cases undergoing CBCT. When interruption of the MC cortical bone was detected in CBCT, the mean distance between the root of the third molars and the canal upper border was 0.56 ± 1.01 mm. Without interruption of the MC cortical bone, the mean distance was 1.12 ± 1.24 mm. In CBCT, interruption of the cortical bone was associated with the presence of radiological parameters, as shown in  Table 1.

Five of the parameters evaluated were statistically associated with interruption of MC cortical bone: a radiolucent band, loss of MC border, change in MC direction, and superimposition with other structures. The nerve was inferior to the third molar root in 38% of CBCT cases, lingual in 31%, buccal in 23%, and between the roots in 7%.

Information provided by CBCT regarding the position of the MC in relation to the roots of the third molar was correlated to the presence of MC cortical bone ( Table 2). A chi-square test was applied to assess the dependent relations, but the low number of observations could bias the analysis. Fisher’s exact test was therefore applied with a significance of 0.05. The absence of cortical and lingual bone was significantly associated with the root position. The correlation between the position of the MC and the ab-sence of cortical bone is reported in  Table 2. Lingual passage of the MC to the roots indicates a higher risk of absence of the MC cortical bone, whereas buccal passage of the nerve correlates moderately with the presence of the MC cortical bone.

**Table 2:**
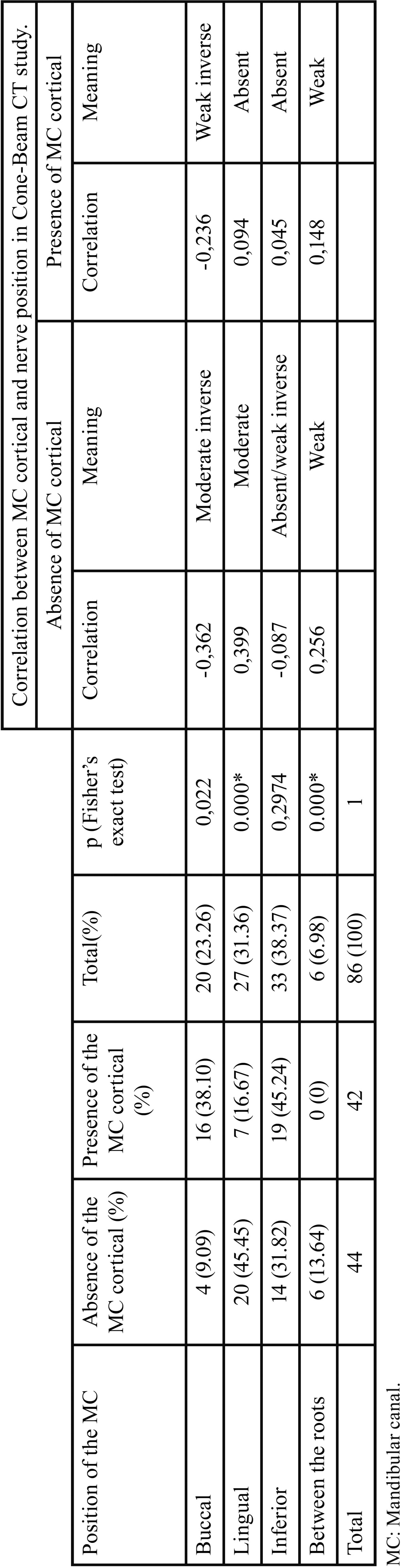
Association between the position of the MC in relation to the tooth root and the presence / absence of cortical bone in Cone-Beam CT study.

## Discussion

**Figure 1:**
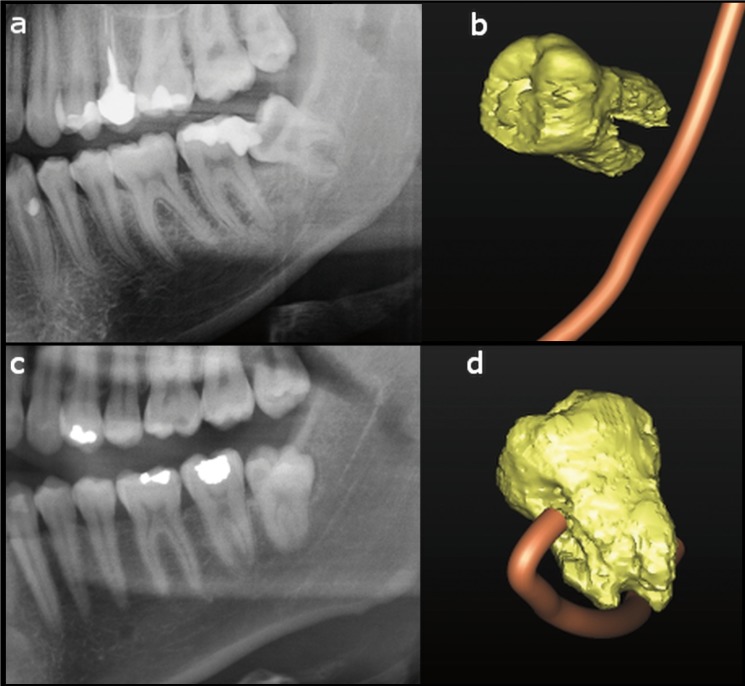
Panoramic radiography magnification of a lower left third molar in a male subject (a). The apex of the root is superimposed on the MC. The CBCT 3D reconstruction of (a) shows no contact between the tooth and the mandibular canal (b). Panoramic radiography magnification of a lower left third molar in a young woman (c). The apex of the root is superimposed on the mandibular canal. In this case the CBCT 3D reconstruction confirm a close contiguity between the tooth and the MC (d).

The biggest risk factor for the occurrence of postoperative neurological damage is contiguity between IAN and the root of the third molar, with the absence of the MC cortical roof. The relationship between the third molar and the MC is not accurately evaluable preoperatively. Two lower left third molars in two different patients are shown in figure 1. While a similar relationship between the tooth and the MC is shown in the panoramic radiographic views, the CBCT radiographic reconstruction shows two different situation. A survey among Australian oral surgeons found that the panoramic sign considered most indicative of contiguity between the canal and the mandibular third molar is MC narrowing, followed by deviation of the canal, disappearance of the edge of the canal, and the presence of a radiolucent band (11). In addition, 31% of surgeons consider only superimposition to be indicative of proximity and 24% of surgeons consider only contact between the apex of the third molar and the MC to be indicative of proximity. In this study, we found no significant association between radiographic indication of contact of the tooth with the MC and the absence of MC cortical bone. In contrast, a high probability of absence of the cortical roof of the MC was recorded when a canal-tooth superimposition was observed in panoramic radiographs.

We found a significant relationship between the depth of inclusion and the presence of the following panoramic signs: loss of MC border, change in MC deviation, and superimposition, indicating that with an increasing depth of inclusion there is an increase of the presence of radiological signs of proximity between the third molar root and the MC.

Also, in the present study, the panoramic signs associated with the absence of the cortical roof of the MC were: radiolucent band, loss of MC border, change in MC direction, superimposition, and MC contact with the root of the third molar. These findings are consistent with those reported by Ghaeminia and colleagues (12), but they did not evaluate three other signs (bifid apex, superimposition, MC contact), as well as with other reports (17-19). Nakagawa and colleagues reported an association between an absence of MC border and an absence of cortical bone in CBCT (16). The most frequent location of the nerve in our study was inferior, followed by a slightly lower frequency of the lingual and buccal positions. The present data are consistent with the literature (12,20-25) ( Table 3). In cases where the MC cortical bone was absent. the nerve was positioned lingually (45% of cases) or inferiorly (32% of cases), while it was rarely observed buccally (9% of cases) or between the root (14% of cases).

**Table 3:**
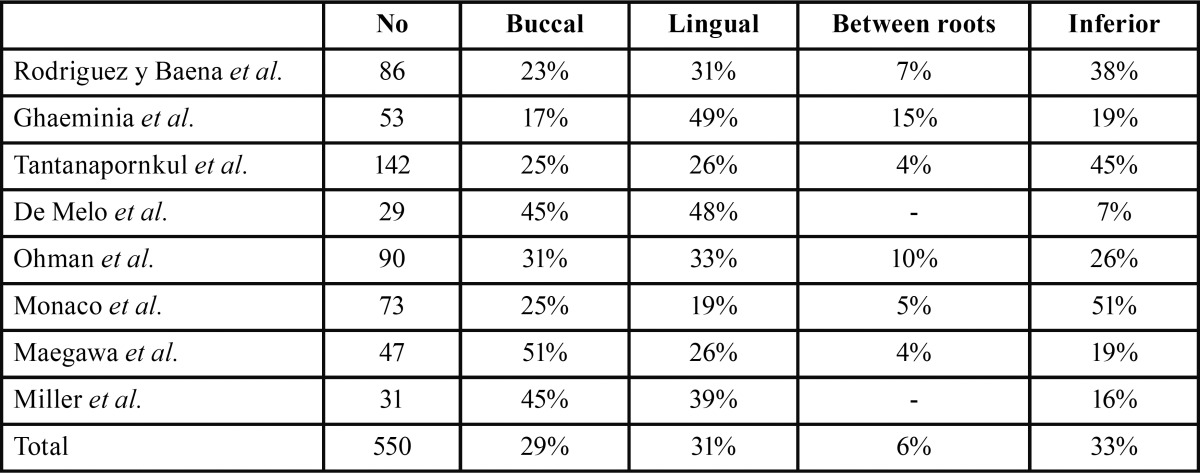
The bucco-lingual position of the mandibular canal with the third molar root as reported in the literature.

The data in the literature are variable in this regard, although there is agreement that the lingual position is most at risk of neurological damage due to the disappearance of MC cortical bone (12,24).

The radiation dose delivered by a Soredex SCANORA™ 3D device is 47 μSv for scans of the mandible (26), which is about twice that delivered with panoramic radiographs, 6-30 times less than a multislice CT, and ~70 times lower than background terrestrial radiation (27).

CBCT, unlike panoramic, radiographs are effective for identifying the actual position of MC with a slightly higher biological risk. Therefore, this test is able to identify cases more at risk, i.e., those in which the MC is lingual, or those lacking cortical protection of the IAN. Such examination, identifying the actual position of the MC, allows for better surgical planning, both in the phase of eventual osteotomy with a rotating burr and during the tooth luxation by identifying movements that could potentially cause compression or laceration of the IAN. Finally, CBCT can be used to identify those cases where the surgery risk is too high to perform a coronectomy (28).

A small study acknowledged by the authors themselves to be too small demonstrated no difference in the incidence of neurological lesions using panoramic or CBCT radiographs for surgical planning, despite having observed a rate of injuries more than double in the panoramic group (29). Another study reported that in 12% of cases the CBCT image changed the surgical approach and treatment plan. Based on this study, evaluation with CBCT in cases at risk allows for better evaluation of the anatomical relationship between the MC and the root of the third molar, revealing the relative position of the root and channel, the presence of cortical bone around the IAN. and the distance between the MC cortical bone and the root of the third molar.

## Conclusions

In cases in which the panoramic radiograph exclude close contiguity between the tooth and the MC examination, CBCT is not indicated. In more complex cases, however, panoramic radiographs should be evaluated according to the signs that differentially contribute to risk quantification. Evaluation with CBCT adds information with a reasonably low radiation dose and therefore seems important for proper risk assessment and to obtain valid informed consent.
